# GLYCINE betaine and seaweed-based biostimulants improved leaf water status and enhanced photosynthetic activity in sweet cherry trees

**DOI:** 10.3389/fpls.2024.1467376

**Published:** 2024-12-20

**Authors:** Sílvia Afonso, Ivo Oliveira, Francisco Guedes, Anne S. Meyer, Berta Gonçalves

**Affiliations:** ^1^ Centre for the Research and Technology of Agro-Environmental and Biological Sciences (CITAB), University of Trás-os-Montes e Alto Douro (UTAD), Vila Real, Portugal; ^2^ Institute for Innovation, Capacity Building and Sustainability of Agri-Food Production (Inov4Agro), University of Trás-os-Montes e Alto Douro, Vila Real, Portugal; ^3^ Cermouros-Cerejas de São Martinho de Mouros, Lda., Resende, Portugal; ^4^ Department of Biotechnology and Biomedicine, Technical University of Denmark, Lyngby, Denmark

**Keywords:** *Prunus avium* L., spray treatments, glycine betaine, *Ecklonia maxima*, leaf gas exchange, leaf metabolites, water status

## Abstract

Sweet cherry is a high-value crop, and strategies to enhance production and sustainability are at the forefront of research linked to this crop. The improvement of plant status is key to achieving optimum yield. Biostimulants, such as glycine betaine (GB) or seaweed-based biostimulants [e.g., *Ecklonia maxima* (EM)], can represent a sustainable approach to improving plant conditions, even under adverse environmental circumstances. Despite their potential, few studies have focused on the effects of GB or EM exogenous application on sweet cherry tree physiology. To address this lack of research, a study was conducted in a Portuguese sweet cherry commercial orchard, using *Lapins* and *Early Bigi* cultivars. Trees were treated with products based on GB and EM at two different concentrations [GB 0.25% (v/v) and GB 0.40% (v/v); EM 0.30% (v/v) and EM 0.15% (v/v)], a combination of the lowest concentrations of both biostimulants (Mix —GB 0.25% and EM 0.15%), and a control group (C) treated with water. Applications were performed over three consecutive years (2019, 2020, and 2021) at three different phenological stages, according to the BBCH scale: 77, 81, and 86 BBCH. Results showed, in general, that the application of biostimulants led to improvements in water status as well as significantly lower values of electrolyte leakage and thiobarbituric acid reactive substances compared to C samples. Additionally, biostimulants reduced pigment loss in the leaves and enhanced their biosynthesis. The Chlorophyll_a_/Chlorophyll_b_ ratio, ranging from 2 to 4, indicated a greater capacity for light absorption and lower stress levels in treated leaves. Soluble sugar and starch content decreased during fruit development in both cultivars and years; however, biostimulants increased these contents, with increments of approximately 15% to 30% in leaves treated with EM. Soluble protein content also showed the same pattern for treated leaves. Biostimulants, especially EM, demonstrated a significant positive effect (*p* ≤ 0.001) on total phenolic content, with increases of approximately 25% to 50% in treated leaves. In conclusion, the application of biostimulants, especially algae-based, significantly improved tree performance by enhancing physiological parameters and stress resilience and could represent a novel approach in fruit production systems.

## 1 Introduction

Climate change, driven by human activities, is one of the greatest challenges of the current century, as it threatens agricultural production and human wellbeing (Bantis and Koukounaras, 2022). The latest Assessment Report of the Intergovernmental Panel on Climate Change (IPCC) projects a continued increase in global average surface temperatures and a decrease in annual precipitation, particularly affecting southern European countries such as Portugal (IPCC and Core Writing Team, 2023). This scenario, coupled with the rising global trade of plants and fruits, driven by population growth and consumer demand for a consistent supply of high-quality products, underscores the urgent need to adopt sustainable agricultural practices. Implementing resource-efficient strategies that align with the principles of the circular economy can help address these challenges (Afonso et al., 2022; Asif et al., 2023; Zulfiqar et al., 2024).

In this context, biostimulants are gaining recognition within the scientific community and businesses alike as a sustainable and eco-friendly alternative to conventional agrochemicals (du Jardin, 2015; Rouphael and Colla, 2020; Basile et al., 2021; Muhie, 2023). When used with good agricultural practices, biostimulants can enhance crop development and productivity and resilience against biotic and abiotic stresses (Moreno-Hernández et al., 2020). Among the seven categories of biostimulants identified by du Jardin (2015), glycine betaine (GB) and seaweed extracts are noteworthy, though their use in cherry tree cultivation remains relatively unexplored (Correia et al., 2020a; Correia et al., 2020b; Serapicos et al., 2022). Given the susceptibility of cherry trees to adverse weather conditions, studying these biostimulants offers a promising approach to improving resilience and productivity, even in challenging environmental circumstances (Rojas et al., 2021; Tudela et al., 2023; Salvadores and Bastías, 2023).

GB, a quaternary amine, plays a role in maintaining membrane integrity under abiotic stress, as it is recognized for its osmoregulatory and osmoprotective functions against drought, salinity, and extreme temperatures (Khalid et al., 2015; Rasheed et al., 2018; Dutta et al., 2019; Fedotova, 2019; Sharma et al., 2023). Its exogenous application in crops has been linked with increased yield potential and improved quality (Shan et al., 2016; Gonçalves et al., 2020; Khalid et al., 2022; Zulfiqar et al., 2022). Similarly, the foliar application of brown macroalgae *Ascophyllum nodosum* and *Ecklonia maxima* seaweed-based biostimulants has been shown to promote plant growth, increase yields, and enhance fruit protein and nutrient content (Jolayemi et al., 2023). Furthermore, it improves leaf health and enhances plant tolerance to abiotic and biotic stress (Parađiković et al., 2019; Ali et al., 2021), due to their complex composition, rich in plant hormones, proteins, amino acids, sugars, vitamins, and phenolic compounds (Gonçalves et al., 2020; Afonso et al., 2022). Despite extensive research on the application of biostimulants in various fruit tree species (Basile et al., 2020; Mones Sardrodi et al., 2022; Serapicos et al., 2022), scientific evidence on the effects of such application on cherry tree performance is scarce. To the best of our knowledge, there is no available information regarding studies on the effects of *E. maxima* seaweed-based biostimulants on cherry tree physiology. Additionally, limited studies are focusing on the application of GB.

In this regard, this study aims to fill this research gap by evaluating the effect of pre-harvest treatments with GB and *E. maxima* seaweed extracts on the physiological and biochemical responses of *Early Bigi* and *Lapins* cherry cultivars grown in northern Portugal.

## 2 Materials and methods

### 2.1 Experimental site and plant material

**Figure 1:**
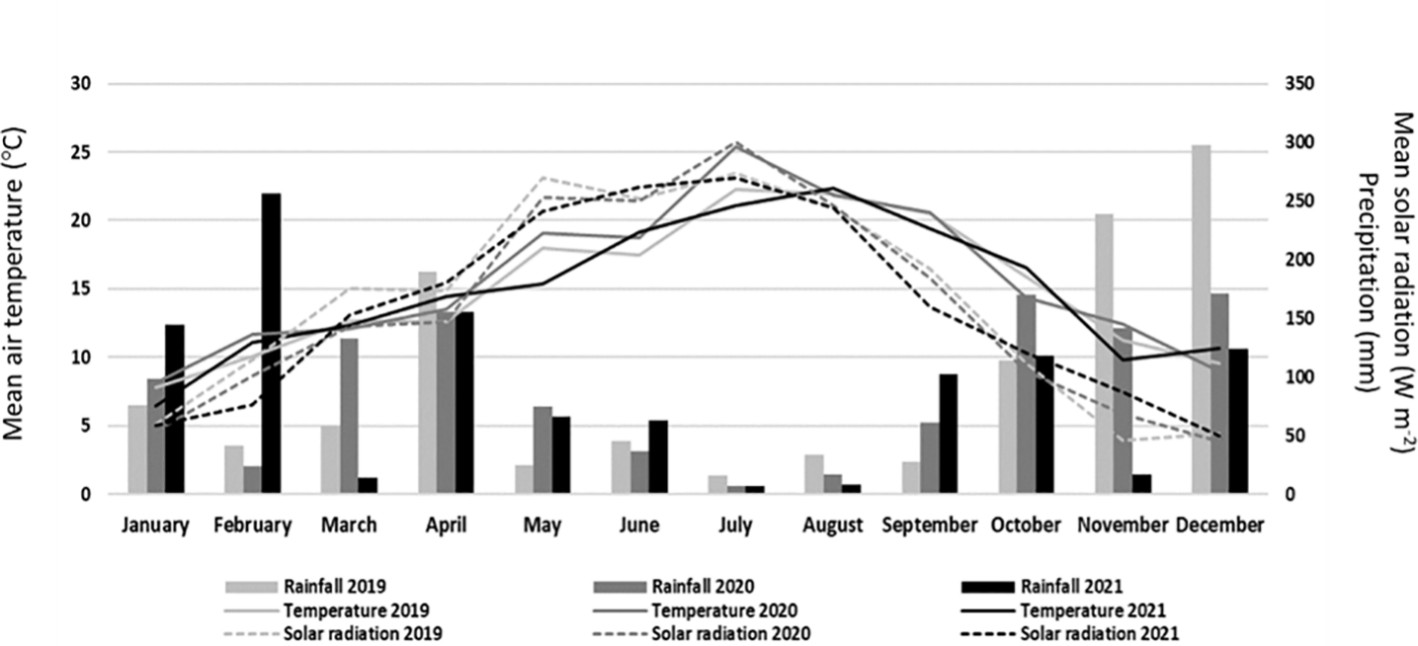
Monthly mean air temperature (°C), precipitation (mm), and mean solar radiation (W m^−2^) in 2019, 2020, and 2021.

The study was carried out at Quinta da Alufinha, municipality of Resende, Portugal (latitude 41°06′ N and longitude 7°54′ W), on a 7-year-old sweet cherry commercial orchard, located at a low altitude (140 m above sea level), in the years of 2019, 2020, and 2021. Trees were trained under a vertical axis system with a spacing of 3.0 m between rows and 2.5 m in the row. Between March and August, trees were drip-irrigated daily, ensuring a uniform water supply. In addition, trees were also periodically fertilized. Standard meteorological variables [air temperature (°CC), rainfall (mm), and solar radiation (W m^−2^)] for the 3 years were recorded by an automatic weather station set up near the experimental site (**Figure 1**). In 2019, the average air temperature between March and May was approximately 1.42°C lower than that of 2020, and 1.09°C higher than that of 2021. Based on the annual precipitation data, the year 2019 recorded the highest annual rainfall (1,163 mm), followed by 2020 (1,091 mm), and 2021 (1,076 mm). Furthermore, the mean solar radiation until May was also higher in 2019 than in the other 2 years. Compared to climate normals available (1971–2000), all 3 years were drier (especially 2021, with 20% less rainfall), while, regarding temperature, all 3 years were all hotter than the 30-year average, namely, 2020 (average hotter by 1°C).

The early cultivar (cv.) *Early Bigi* and the late cv. *Lapins*, both grafted onto “Santa Lucia 64”, were selected for this trial, due to its economic importance of this region.

### 2.2 Experimental design and treatments

Eight trees from each cultivar, homogeneous, and in good phytosanitary conditions, were chosen for each treatment, resulting in a total of 48 trees per cultivar. Six treatments (expressed as % of volume/volume) were foliar sprayed, using a mechanical sprayer, and they included two concentrations of GB (97%) (GB 0.25% and GB 0.40%), two concentrations of *E. maxima* seaweed-based biostimulants (EM 0.30% and EM 0.15%), a combination of the lowest concentrations of both biostimulants (Mix —GB 0.25 and EM 0.15), and a control (C) group (water). Foliar treatments were repeated at three different dates during the phenological stages of the cherry tree, according to the BBCH scale [Biologische Bundesantalt, Bundessortenamt und Chemische Industrie (Fadon et al., 2015)]: stage 77 (70% of final fruit size), stage 81 (beginning of fruit coloring), and stage 86 (coloring advanced, 3 days before fruit harvesting). To achieve more accurate results, treatments (**Table 1**) were applied for three consecutive years (2019, 2020, and 2021), in a windless morning (**Table 2**).

**Table 1:** Experimental treatments and their corresponding concentrations.

Treatment code	Product and concentration
C	Water only
GB 0.25%	Glycine betaine applied at a concentration of 0.25% (v/v)
GB 0.25%	Glycine betaine applied at a concentration of 0.40% (v/v)
EM 0.15%	*Ecklonia maxima* applied at a concentration of 0.15% (v/v)
EM 0.30%	*Ecklonia maxima* applied at a concentration of 0.30% (v/v)
MIX	Glycine betaine at a 0.25% (v/v) + *Ecklonia maxima* 0.15% (v/v)

**Table 2:** Foliar application dates of biostimulants on sweet cherry trees over the 3 years of trial.

		BBCH scale		
		77	81	86
		Phenological stage		
		70% of final fruit size	Beginning of fruit coloring	Coloring advanced (3 days before fruit harvesting)
Year	Cultivar			
2019	*Early Bigi*	11 April	19 April	30 April
	*Lapins*	11 April	16 May	24 May
2020	*Early Bigi*	1 April	11 April	16 April
	*Lapins*	1 April	4 May	12 May
2021	*Early Bigi*	6 April	15 April	24 April
	*Lapins*	6 April	11 May	31 May

Healthy, fully expanded, sun-exposed, and mature leaves in a similar position were carefully chosen for the assessment of gas exchange, electrolyte leakage (EL), and relative water content (RWC). These assessments were carried out at solar noon in both cultivars, 3 days after the application of the biostimulants, for the years 2019 and 2021. Additionally, about seven leaves per tree and treatment were collected for biochemical analyses and were immediately frozen in liquid nitrogen, ground to a fine powder, and then stored at −80°C for further analysis. In 2020, despite the foliar applications of the treatments being carried out, it was impossible to conduct these assessments due to the COVID-19 pandemic.

### 2.3 Leaf gas exchange

Leaf gas exchange measurements were accomplished using a portable infrared gas analyzer system (LC Pro+, ADC, Hoddesdon, UK). The IRGA was equipped with a 2. 5-cm^2^ leaf chamber (ADC-PLC) and operated in open mode. Incident photosynthetic photon flux density (PPFD) on the leaves was always greater than 1,500 μmol m^−2^ s^−1^. The measurements were performed on cloudless days, under natural light conditions, and at solar noon. Through the formulas proposed by von Caemmerer and Farquhar (1981), the net photosynthetic rate (A, μmol m^−2^ s^−1^), transpiration rate (E, mmol m^−2^ s^−1^), stomatal conductance (g_s_, mmol m^−2^ s^−1^), and intercellular CO_2_ concentration (Ci, µmol mol^−1^) were calculated. The intrinsic water use efficiency was calculated as the ratio of A/g_s_ (A/g_s_, µmol mol^−1^), to dismiss the potential effects of air humidity and temperature on transpiration (Iacono et al., 1998). Measurements were only performed in the year 2019 (for cv. *Early Bigi* on April 14, April 22, and May 3, and for cv. *Lapins* on April 14, May 19, and May 27), as the portable infrared gas analyzer system was unavailable in 2021. Results are expressed as the mean ± standard deviation (SD) of eight replicates.

### 2.4 Leaf water status

For water status analysis, detached sweet cherry leaves were immediately placed into airtight containers with the petiole facing downwards. Fresh weight (FW, g) was measured, and after immersing leaf petioles in demineralized water for 48 h in the dark at 4°C, turgid weight (TW, g) was measured. Dry weight (DW, g) was determined after drying at 70°C until a constant weight was achieved. Leaf area (LA, cm^2^) was also measured using the WinDias image analysis system (Delta-T Devices Ltd., Cambridge, UK). Furthermore, the RWC was calculated as:


$$\text{RWC }\left(\%\right)=\frac{\left(FW-DW\right)}{\left(TW-DW\right)}\;\text{x}\;100$$

Leaf mass per unit area (LMA) (g m^−2^) was calculated by the ratio between leaf area and DW. Results are expressed as the mean ± standard error (SE) of eight replicates.

### 2.5 Metabolite composition determination

#### 2.5.1 Photosynthetic pigments

The chlorophylls and carotenoids were extracted with 80% acetone by weighing 25 mg into a screw tube and adding 4 mL of 80% acetone solution. The mixture was then homogenized using a vortex and sonicated for 5 min at 30 Hz. Subsequently, the mixture was centrifuged at 4,000 rpm at 4°C for 10 min. Next, 200 μL of each sample was transferred to a 96-well microplate, and absorbance readings were taken at 470, 645, and 663 nm against a blank. Chlorophyll a (Chl_a_), chlorophyll b (Chl_b_), and total chlorophyll [Chl_(a+b_)] were determined according to Arnon (1949) and Sestak et al. (1971), and total carotenoids (Car_total_) were determined according to Lichtenthaler (1987). Additionally, ratios Chl_a_/Chl_b_ and Chl_(a+b)_/Car_total_ were also determined. All procedures were performed under dim light to prevent photodegradation, isomerization, and structural changes of photosynthetic pigments, and results were expressed as mg g^−1^ DW, as the mean ± SD of eight replicates.

#### 2.5.2 Total soluble sugars and starch

Total soluble sugars (SS) were quantified using the spectrophotometry method described by Irigoyen et al. (1992). Samples were heated in ethanol/water (80/20, v/v) for 1 h at 80°C. TSS were measured, at 625 nm, after the alcoholic extract reacted with fresh anthrone in a boiling water bath for 10 min. The soluble fractions were then separated from the solid fraction. Starch (St) was extracted from the same solid fraction by heating it in 30% perchloric acid for 1 h, at 60°C, following the method of Osaki et al. (1991). The St concentration was determined by the anthrone method, as previously described, and both SS and St are expressed as mg g^−1^ DW, using glucose as a standard, presented as the mean ± SD of eight replicates.

#### 2.5.3 Soluble proteins

Total soluble protein (TP) quantification followed the Bradford (1976) method. Total soluble proteins were extracted using a phosphate buffer (pH 7.5) containing 0.1 mM ethylenediaminetetraacetic acid (EDTA), 100 mM phenyl-methylsulfonyl fluoride (PMSF), and 2% (w/v) polyvinylpyrrolidone (PVP), followed by centrifugation at 12,000×*g* at 4°C, for 30 min. Absorbance was measured at 595 nm using bovine serum albumin (BSA) as a standard. The results, expressed as mg g^−1^ DW, were the mean ± SD of eight replicates.

#### 2.5.4 Total phenolics

For the quantification of total phenolics, a previous extraction was performed, as previously described by Serapicos et al. (2022): 40 mg of each sample (DW) was added to 1.5 mL of 70% (v/v) methanol, mixed thoroughly on a vortex for 30 min, and centrifuged at 5,000 rpm at 4°C for 15 min. The supernatant was collected into a 10-mL volumetric flask. This procedure was repeated three more times, with the final volume adjusted to 10 mL using methanol. Total phenolics were then determined using the method by Singleton and Rossi (1965) and Dewanto et al. (2002), with some modifications. In each well of a 96-well microplate, 20 µL of each leaf extract, 100 µL of Folin-Ciocalteu reagent (1:10 in bidistilled water), and 80 µL of 7.5% Na_2_CO_3_ were mixed. The microplate was incubated in the dark for 15 min at 45°C. Absorbance values were measured at 765 nm against a blank. The colorimetric response of total phenols measurements was compared to a standard curve based on gallic acid, and the results were expressed as mg gallic acid equivalents (GAE) g^−1^ DW, as the mean ± SD of eight replicates.

### 2.6 Cell membrane damage

#### 2.6.1 Electrolyte leakage

Leaf EL was measured to assess cell membrane permeability, based on the method described by Mena-Petite et al. (2001) with modifications. After collecting the leaves, they were washed in deionized water to remove surface ions. Foliar discs with a diameter of 0.8 cm were then punched out from each leaf and placed in test tubes with 10 mL of deionized water. Incubation at 25°C was carried out for 24 h on a rotary shaker. After incubation, the electrical conductivity of the solution (CE_1_) was measured using a conductivity meter (Mettler Toledo). The samples were autoclaved at 120°C for 20 min, and a new reading of electrical conductivity (CE_2_) was taken, after cooling to 25°C. The EL (%) was calculated as follows:


$$\text{EL}=\frac{CE1}{CE2}\text{x}\;100.$$

The values are presented as the mean ± SD of eight replicates.

#### 2.6.2 Lipid peroxidation

To assess cell membrane lipid peroxidation, thiobarbituric acid reactive substances (TBARS) were quantified according to Hodges et al. (1999), with some adaptations. Briefly, the lyophilized samples were frozen in liquid nitrogen and ground in 20% (w/v) trichloroacetic acid with mortar and pestle. The absorbance of the supernatant was measured at 532 and 600 nm. TBARS were calculated using the malondialdehyde (MDA) extinction coefficient of 155 mM cm^−1^. Lipid peroxidation was expressed in mmol MDA equivalents g^−1^ DW, as the mean ± SD of eight replicates.

### 2.7 Statistical analysis

The statistical analysis was conducted using the SPSS V.27 software (SPSS-IBM, Corp., Armonk, New York, USA). After testing for analysis of variance (ANOVA) assumptions, namely, the homogeneity of variances with Levene’s mean test and normality with the Shapiro–Wilk’s test, statistical differences among treatments within each variety and year were evaluated by one-way ANOVA, followed by the *post-hoc* Tukey’s test. To assess the effects of treatment, year, phenological stage, and their interactions, a multivariate analysis of variance (MANOVA) was performed using Pillai’s trace statistic in SPSS V.27 software. Differences were considered statistically significant at a significance level of *p* ≤ 0.05.

Correlations between measured gas exchange parameters were assessed using Pearson correlation coefficients.

Data are presented as the mean ± SD of eight replicates, and the results are presented by dry weight, for photosynthetic pigments. For leaf water status, total soluble sugars, starch and protein, total phenolics, and cell membrane damage, data are presented as the mean ± SD of eight replicates.

## 3 Results and discussion

### 3.1 Leaf gas exchange parameters

The application of biostimulants affected the two tested cultivars differently regarding leaf gas exchange parameters (**Tables 3**, **4**). For cv. *Early Bigi*, the general trend in leaf gas exchange parameters showed an increase as the season progressed (**Table 3**). The effect of spraying biostimulants appears to have some influence, as C leaves always presented the lowest values, even though significant effects of treatments were not recorded on all sampling dates. Indeed, at the first sampling date, significant effects were not recorded for Ci and A/g_s_, while for other parameters, higher values were observed in trees treated with GB 0.25%, even though these values were similar to other treatments (for A, similar to GB 0.25% and Mix treatments; for E and g_s_, similar to EM 0.3% and Mix). In the second sampling moment, a different trend was found, with differences between treatments only recorded for Ci, and only when comparing EM 0.3% to C leaves. At fruit harvest, significant changes were only recorded for E and g_s_. For the former parameter, lower values were recorded for the C treatment, while the transpiration rate increased in all other treatments, namely, in the lower dosages of EM and GB. The effects recorded on stomatal conductance follow a similar pattern, although significant differences were only observed between the C and GB 0.25% treatments.

**Table 3:** Photosynthetic rate (A), transpiration rate (E), stomatal conductance (g_s_), intercellular CO_2_ concentration (Ci), and intrinsic water use efficiency (A/g_s_) recorded in leaves of cv. *Early Bigi*, recorded in 2019.

cv. *Early Bigi*	Date	C	EM 0.15%	EM 0.30%	GB 0.25%	GB 0.40%	Mix	p
A (µmol m^−2^ s^−1^)	14/4	8.69 ± 0.87b	9.82 ± 0.89b	9.75 ± 0.83b	11.11 ± 1.53a	12.8 ± 1.54a	10.59 ± 1.31a	***
	22/4	6.39 ± 1.71	12.21 ± 5.12	8.53 ± 2.85	13.84 ± 4.19	9.02 ± 2.31	14.21 ± 4.82	n.s.
	3/5	7.56 ± 1.55	12.31 ± 5.29	11.51 ± 2.26	12.55 ± 2.26	10.42 ± 3.45	11.39 ± 2.82	n.s.
E (mmol m^−2^ s^−1^)	14/4	1.67 ± 0.17c	2.94 ± 0.89b	1.81 ± 0.24c	2.44 ± 0.52b	3.79 ± 0.31a	2.81 ± 0.18b	***
	22/4	1.72 ± 0.24	2.01 ± 0.22	1.94 ± 0.14	1.80 ± 0.08	1.72 ± 0.33	2.02 ± 0.22	n.s.
	3/5	3.14 ± 0.92b	5.36 ± 0.82a	4.59 ± 0.67ab	5.53 ± 0.50a	4.18 ± 0.51ab	4.79 ± 0.91a	***
g_s_ (mmol m^−2^ s^−1^)	14/4	106.29 ± 17.23b	124.78 ± 48.41b	156.84 ± 20.49ab	108.40 ± 9.75b	190.01 ± 25.99a	167.83 ± 29.15a	***
	22/4	154.73 ± 38.86	195.60 ± 43.36	209.04 ± 23.06	174.08 ± 9.13	206.57 ± 43.58	194.78 ± 31.04	n.s.
	3/5	158.52 ± 14.49b	213.81 ± 60.47ab	228.79 ± 94.01ab	349.54 ± 71.52a	259.58 ± 75.89ab	210.78 ± 74.06b	*
Ci (µmol mol^−1^)	14/4	219.64 ± 49.54	228.79 ± 9.89	271.20 ± 29.57	222.69 ± 45.71	253.58 ± 12.81	270.52 ± 29.57	n.s.
	22/4	228.99 ± 56.22b	273.79 ± 77.91ab	348.29 ± 52.11a	337.34 ± 29.89ab	324.22 ± 11.54ab	251.97 ± 76.98ab	**
	3/5	265.83 ± 11.19	301.51 ± 29.86	302.27 ± 42.67	324.14 ± 73.05	283.09 ± 15.02	281.52 ± 52.58	n.s.
A/g_s_ (µmol mol^−1^)	14/4	81.13 ± 9.24a	79.54 ± 7.07a	35.21 ± 15.75c	102.86 ± 11.32a	67.92 ± 9.99b	67.19 ± 9.59b	***
	22/4	41.14 ± 5.69b	63.86 ± 29.57ab	40.29 ± 13.65ab	80.39 ± 26.97a	44.57 ± 10.34ab	72.83 ± 29.57ab	**
	3/5	48.38 ± 14.03	57.34 ± 8.13	50.14 ± 18.63	35.42 ± 21.37	46.85 ± 13.51	57.12 ± 15.59	n.s.

Data are mean ± SD of eight replicates. Different lowercase letters represent significant differences between treatments. The absence of letters indicates no significant differences between treatments (n.s., not significant). Asterisks represent significant differences (***p ≤ 0.001; **p ≤ 0.01; *p ≤ 0.05).

**Table 4:** Photosynthetic rate (A), transpiration rate (E), stomatal conductance (g_s_), intercellular CO_2_ concentration (Ci), and intrinsic water use efficiency (A/g_s_) recorded in leaves of cv. *Lapins*, recorded in 2019.

cv. *Lapins*	Date	C	EM 0.15%	EM 0.30%	GB 0.25%	GB 0.40%	Mix	p
A (µmol m^−2^ s^−1^)	21/4	10.34 ± 1.92	8.22 ± 2.21	9.36 ± 2.28	9.22 ± 1.06	10.96 ± 2.43	10.06 ± 0.73	n.s.
	19/5	7.68 ± 2.51ab	7.38 ± 1.96b	10.54 ± 2.02a	10.97 ± 2.51a	11.15 ± 3.29a	12.19 ± 1.84a	*
	27/5	6.40 ± 1.20b	10.67 ± 1.96a	7.13 ± 1.82b	7.79 ± 1.59a	10.48 ± 1.17a	8.47 ± 1.88ab	***
E (mmol m^−2^ s^−1^)	21/4	3.00 ± 0.45	2.82 ± 0.42	2.98 ± 0.29	2.88 ± 0.09	3.13 ± 0.50	3.23 ± 0.15	n.s.
	19/5	3.67 ± 0.82bc	4.78 ± 0.78ab	5.39 ± 1.09a	4.42 ± 0.55a	5.74 ± 1.07a	3.52 ± 0.64c	***
	27/5	2.95 ± 0.81b	4.49 ± 0.54a	3.35 ± 1.04ab	3.87 ± 0.70ab	4.45 ± 0.81a	3.06 ± 0.36ab	**
g_s_ (mmol m^−2^ s^−1^)	21/4	132.39 ± 27.16	115.25 ± 23.17	132.26 ± 21.34	114.94 ± 3.71	163.19 ± 35.07	144.99 ± 11.69	n.s.
	19/5	91.23 ± 29.62b	140.74 ± 41.17ab	199.96 ± 70.59ab	213.00 ± 48.96	251.12 ± 87.59a	213.76 ± 59.87a	**
	27/5	65.06 ± 9.44b	134.02 ± 27.58a	83.43 ± 33.42ab	121.92 ± 24.01ab	123.74 ± 37.52ab	116.58 ± 47.56ab	*
Ci (µmol mol^−1^)	21/4	244.41 ± 18.97	270.12 ± 19.97	267 ± 24.94	254.04 ± 11.04	255.89 ± 34.40	262.34 ± 18.72	n.s.
	19/5	234.18 ± 17.66b	287.34 ± 23.96a	283.32 ± 19.11a	279.25 ± 16.23a	288.12 ± 18.45a	262.31 ± 22.01ab	***
	27/5	214.63 ± 28.17	217.43 ± 38.84	223.53 ± 26.13	271.33 ± 23.59	218.94 ± 24.89	240.06 ± 52.34	n.s.
A/g_s_ (µmol mol^−1^)	21/4	79.16 ± 11.69	71.12 ± 9.77	69.87 ± 9.34	80.31 ± 9.73	71.57 ± 20.35	69.97 ± 8.85	n.s
	19/5	83.92 ± 9.89a	53.44 ± 11.01b	55.25 ± 10.22b	51.86 ± 7.19b	45.72 ± 9.82b	59.39 ± 12.65b	***
	27/5	98.81 ± 14.71	89.15 ± 22.06	89.83 ± 16.59	59.35 ± 11.66	89.04 ± 14.05	81.12 ± 29.73	n.s

Data are mean ± SD of eight replicates. Different lowercase letters represent significant differences between treatments. The absence of letters indicates no significant differences between treatments (n.s., not significant). Asterisks represent significant differences (***p ≤ 0.001; **p ≤ 0.01; *p ≤ 0.05).

Regarding cv. *Lapins*, and in contrast to cv. *Early Bigi*, there is no noticeable major trend in leaf gas exchange as the season progresses. For instance, A is lower at harvest than during the first sampling, whereas E is higher (**Table 4**). Regarding the application of biostimulants, no significant effects were recorded on the first sampling date, as the data were similar among treatments for all analyzed parameters. On the second sampling date, significant differences were noted for all leaf gas exchange parameters, with lower values recorded for C in almost all of them, except for A/g_s_. The influence of the applied treatments was also evident at harvest, as only Ci and A/g_s_ values were statistically similar across all samples (**Table 4**). Indeed, values of A, E, and g_s_ were significantly different among treatments, with higher values recorded for EM 0.15% for A (significantly different from C and EM 0.30%), E (significantly different from C), and g_s_ (also significantly different from C).

Some correlations were found between the measured gas exchange parameters. Indeed, for cv. *Early Bigi*, the photosynthetic rate (A) presented correlations with Ci (*r* = −0.595, *p* ≤ 0.001), g_s_ (*r* = 0.220, *p* = 0.043), and E (*r* = 0.215, *p* = 0.048). Similarly, for cv. *Lapins*, correlations were also found, namely, for A and g_s_ (*r* = 0.697, *p* ≤ 0.001) and between A and E (*r* = 0.433, *p* ≤ 0.001). These relationships have been previously reported in sweet cherries (Gonçalves et al., 2005, 2007; Correia et al., 2020b; Houghton et al., 2023). Furthermore, the positive effects on leaf gas exchange parameters due to the application of biostimulants have been documented in various crops. For example, the application of *E. maxima* has sowed beneficial effects in melon, cucumber, and tomato (Lefi et al., 2023); *Cucurbita pepo* L (Rouphael et al., 2017); or *Vigna unguiculata* (Gyogluu Wardjomto et al., 2023). The use of GB as a biostimulant has improved leaf gas exchange parameters in olive (Denaxa et al., 2020), *Lactuca sativa* (Lin et al., 2020) or *Solanum lycopersicum* (Annunziata et al., 2019).

This improvement in leaf gas exchange parameters can be linked to several factors. Enhanced photosynthesis may lead to increased dry matter accumulation (Carvalho et al., 2019; Mateus-Cagua and Rodríguez-Yzquierdo, 2019), by enhancement of mineral nutrient availability and uptake (Caruso et al., 2019), plant hormone regulation, and an increase in metabolites that benefit the electron transport chain (Sorrentino et al., 2022). Furthermore, biostimulants can also potentially improve water-use efficiency in plants (Burghardt and Riederer, 2006; Jiménez-Arias et al., 2022), increasing turgor pressure in leaf guard cells and enhancing gaseous exchange attributes.

### 3.2 Leaf water status

**Figure 2:**
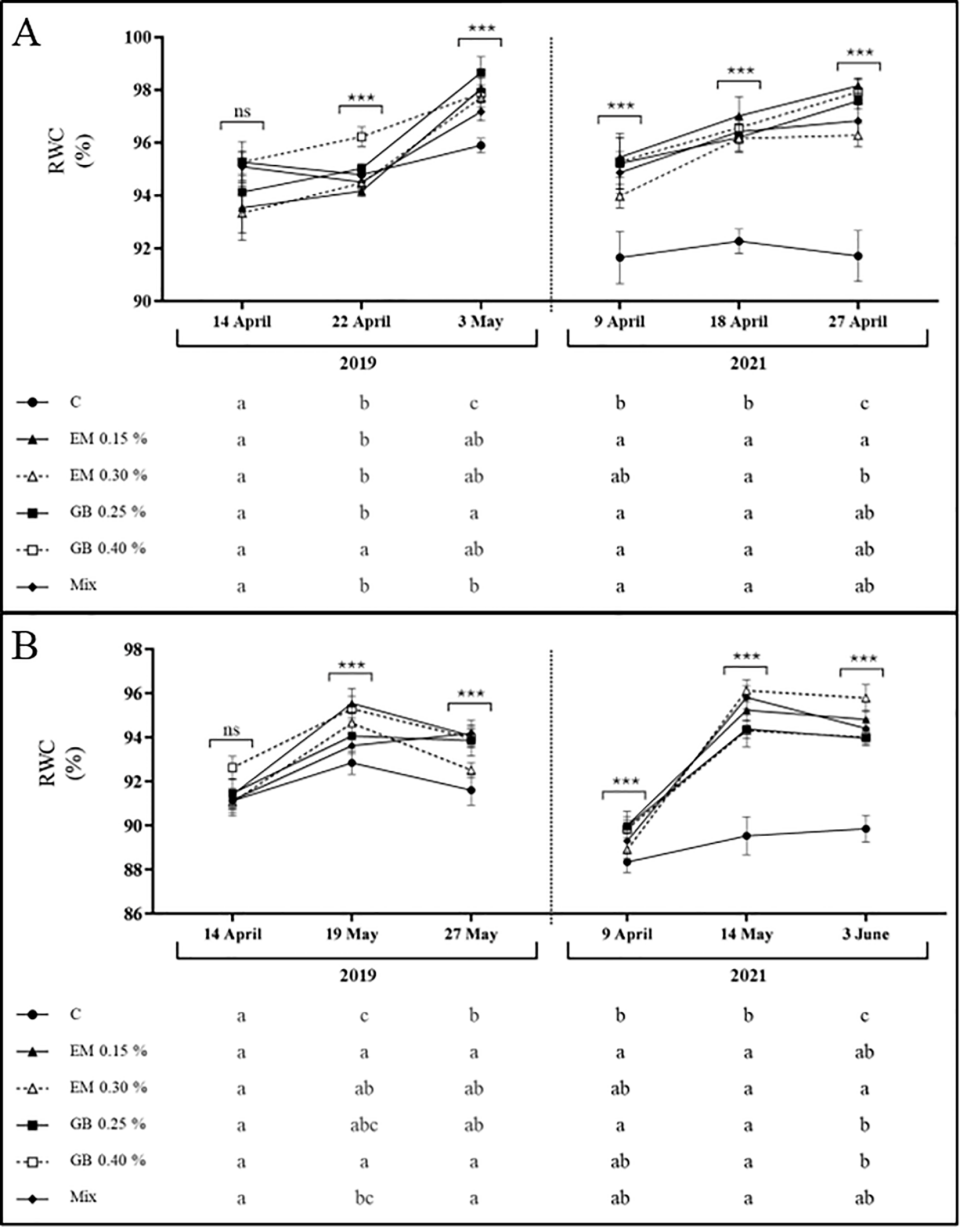
Relative water content (RWC, %) of cv. *Early Bigi* **(A)** and cv. *Lapins* **(B)** cherry leaves after spray treatment application in 2019 and 2021. Data are presented as mean ± SD of eight replicates. Different lowercase letters indicate significant differences between treatments. The absence of letters indicates no significant differences between treatments (n.s., not significant). Asterisks represent significant differences (***p ≤ 0.001).

The leaf water status of both cultivars was affected by different factors (years, phenological stage, treatment, and their interaction), although in different ways (**Supplementary Table S1**). Regarding cv. *Early Bigi*, significant influence of the RWC was recorded for all factors, apart from the year and the interaction of treatment, year, and phenological stage, while for cv. *Lapins*, influence was found for all factors and their interaction. For cv. *Early Bigi*, the RWC trend was similar in both 2019 and 2021, with an increase in values during fruit development (**Figure 2**). However, in 2019, significant changes were more noticeable in the last sampling date (at fruit harvest), with differences in treatments C and Mix, both with lower values, while treatment GB 0.25% had higher values. In 2021, significant differences were observed in all sampling moments, with a clear difference recorded for the C treatment (always lower values), while higher values were observed when using the EM 0.15% treatment (even though it does not differ significantly from other treatments, except for the abovementioned C). For cv. *Lapins*, there is also a similar trend in both years, with an increase of RWC from the first to the second sampling date. However, the values recorded at harvest differed: in 2019, there was a reduction, while in 2021, the water content remained almost the same (**Figure 2**). In 2019, and at the first sampling date, no differences were observed between treatments, but in the following analyses, C samples recorded the lowest RWC, with leaves from EM 0.15% sprayed trees recording the higher values (even though statistically similar to other treatments). In 2021, C leaves recorded the lowest values even at the first sampling date, with leaves from the EM 0.30% treatment presenting increased water content. Water stress can influence RWC values, which can result in growth limitations and changes in physiological and metabolic processes (Irigoyen et al., 1992), with the benefits of high RWC arising from greater resistance of cell walls and their ability to endure tissue destruction or mechanical damage caused by dehydration. However, in the present study, no water stress situation appears to be occurring, as RWC values are usually above 90%, suggesting sufficient drip irrigation, except for those recorded for the C treatment, in 2021. Even so, the application of both biostimulants on either cultivar results in increased RWC values, with a more pronounced effect for cv. *Early Bigi*, in 2021, a year that was hotter and dryer than 2019. The use of GB or algae-based biostimulants has demonstrated positive effects on sweet cherry, as reviewed by Afonso et al. (2022), including an increase in RWC (Correia et al., 2020b; Serapicos et al., 2022), corroborating our findings.

**Figure 3:**
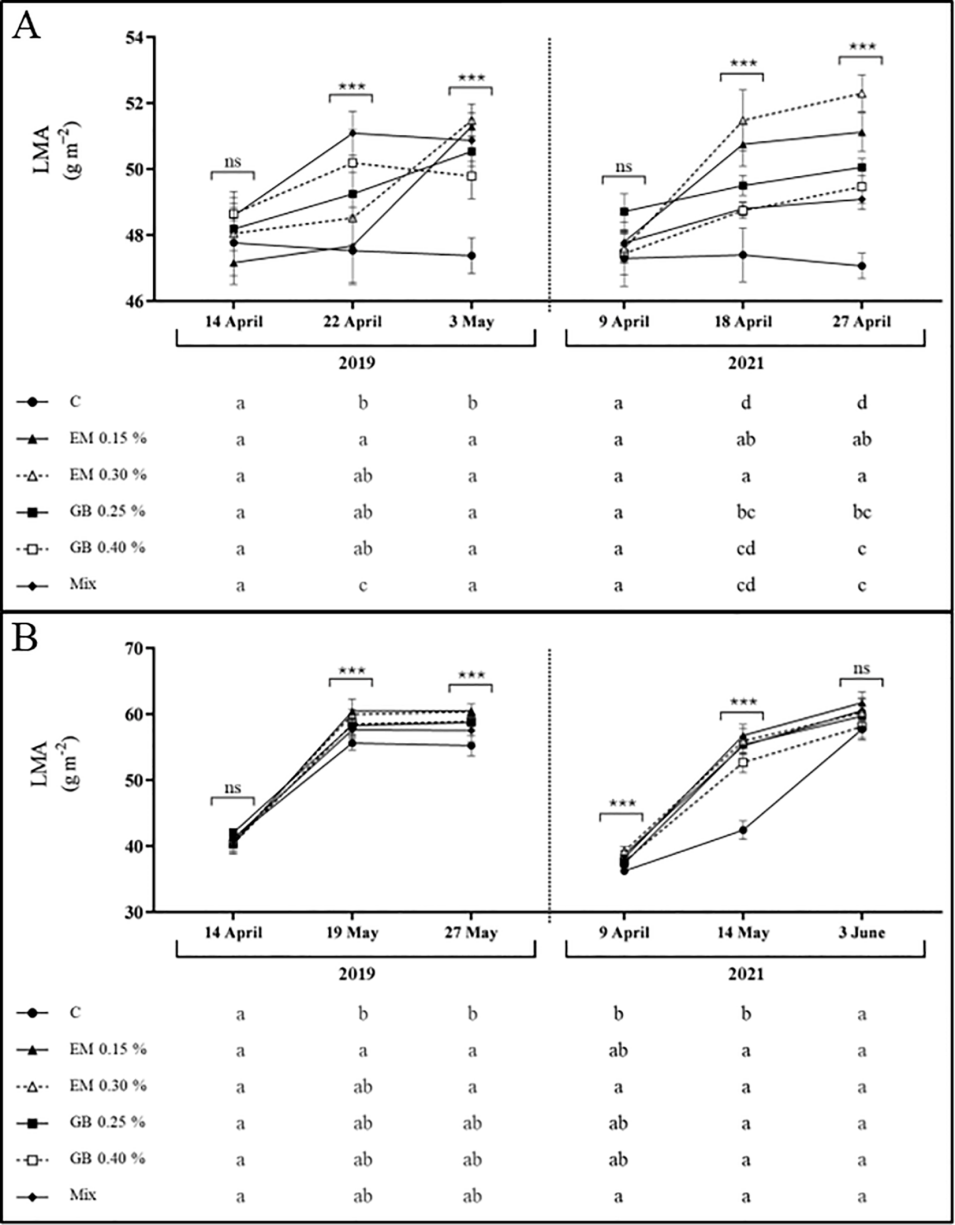
Leaf mass per unit area (LMA, g m^−2^) of cv. *Early Bigi* **(A)** and cv. *Lapins* **(B)** cherry leaves after spray treatment application in 2019 and 2021. Data are presented as mean ± SD of eight replicates. Different lowercase letters represent significant differences between treatments. The absence of letters indicates no significant differences between treatments (n.s., not significant). Asterisks represent significant differences (***p ≤ 0.001).

Data for leaf mass per unit area (LMA) show, again, different behavior for the two cultivars under study (**Figure 3**). This variable was affected by all factors, with the exception of the interaction of the treatment, year, and phenological year for both cultivars, and the interaction of treatment and year was only recorded for cv. *Lapins* (**Supplementary Table S1**). Values recorded for cv. *Early Bigi* shows an overall increase of LMA with fruit development, in both years of study. Interestingly, no differences in LMA were recorded at the first sampling date in either year. However, at subsequent stages, C leaves exhibited lower LMA compared to the other treatments, particularly EM 0.15% and GB 0.40%. Regarding cv. *Lapins*, a similar trend was observed, with an increase in LMA with fruit development. Nonetheless, C leaves consistently had lower values, except for the first sampling date of 2019 and at harvest, in 2021. In contrast, algae-based biostimulants, specifically the EM 0.15% treatment, resulted in increased LMA throughout all sampling dates. High values of LMA have been associated with a higher density of mesophyll or thickness of total lamina, reflecting an increased tolerance to adverse conditions (Centritto, 2002; de la Riva et al., 2016), with improved photosynthetic capacity often related to higher LMA (Reich et al., 2000).

### 3.3 Metabolite composition determination

#### 3.3.1 Photosynthetic pigments

Leaf photosynthetic pigment content was influenced by the application of either biostimulant (*p* ≤ 0.001) (**Tables 5**, **6**, and **Supplementary Table S1**) yet remained within the range recorded for sweet cherry leaves (Gonçalves et al., 2006, 2008). Conversely, the effect of the factors (year, phenological stage, and treatment and their interactions) presented a complex behavior depending on the studied cultivar (**Supplementary Table S1**). Furthermore, chlorophyll content increased as the season progressed, a pattern previously recorded in sweet cherries (Flore and Layne, 1999; Sytykiewicz et al., 2013).

**Table 5:** Total chlorophyll [Chl_(a+b_)], carotenoids (Car), and related ratios [Chl_a_/Chl_b_, Chl_(a+b)/_Car] of cv. *Early Bigi* leaves in 2019 and 2021.

cv. *Early Bigi*		Date	C	EM 0.15%	EM 0.30%	GB 0.25%	GB 0.40%	Mix	p
Chl_(a+b)_ (µg g^−1^ DW)	2019	14/4	3.13 ± 1.54 b	3.25 ± 0.23 b	3.58 ± 1.02 b	5.19 ± 0.91 a	3.78 ± 0.79 ab	4.58 ± 1.07 ab	***
		22/4	3.33 ± 0.23 c	3.80 ± 1.11 bc	3.35 ± 0.38 c	5.41 ± 0.60 a	3.92 ± 0.69 bc	4.69 ± 0.94 ab	***
		3/5	3.42 ± 0.23 c	3.66 ± 0.79 bc	3.45 ± 0.34 c	5.64 ± 0.50 a	4.32 ± 0.36 b	5.33 ± 0.48 a	***
	2021	9/4	2.15 ± 0.53 c	2.24 ± 0.40 c	2.47 ± 0.35 bc	3.00 ± 0.47 ab	3.02 ± 0.34 ab	3.70 ± 0.66 a	***
		18/4	2.32 ± 0.49 c	2.47 ± 0.28 c	2.64 ± 0.44 bc	3.02 ± 0.41 b	3.13 ± 0.30 b	3.94 ± 0.23 a	***
		27/4	2.89 ± 0.41 b	3.43 ± 0.74 ab	3.41 ± 0.69 ab	4.14 ± 0.60 a	3.20 ± 0.48 b	3.31 ± 0.63 ab	**
Car (µg g^−1^ DW)	2019	14/4	0.82 ± 0.41	0.77 ± 0.08	0.84 ± 0.19	1.11 ± 0.17	0.93 ± 0.24	1.00 ± 0.18	n.s.
		22/4	0.76 ± 0.10 c	0.85 ± 0.27 bc	0.79 ± 0.07 bc	1.14 ± 0.12 a	0.96 ± 0.21 abc	1.02 ± 0.14 ab	***
		3/5	0.79 ± 0.21 b	0.80 ± 0.06 b	0.84 ± 0.05 b	1.09 ± 0.15 a	1.06 ± 0.11 a	1.11 ± 0.08 a	***
	2021	9/4	0.45 ± 0.10 c	0.70 ± 0.17 a	0.56 ± 0.10 abc	0.70 ± 0.06 a	0.66 ± 0.08 ab	0.53 ± 0.10 bc	***
		18/4	0.47 ± 0.09 c	0.73 ± 0.05 a	0.60 ± 0.05 abc	0.69 ± 0.10 ab	0.63 ± 0.07 ab	0.58 ± 0.15 bc	***
		27/4	0.49 ± 0.05 b	0.62 ± 0.11 ab	0.61 ± 0.11 ab	0.66 ± 0.11 a	0.56 ± 0.07 ab	0.61 ± 0.13 ab	**
Chl_a_/Chl_b_	2019	14/4	3.03 ± 0.11	3.03 ± 0.20	3.07 ± 0.13	3.09 ± 0.16	3.01 ± 0.21	3.09 ± 0.26	n.s.
		22/4	3.04 ± 0.51	2.77 ± 0.91	3.08 ± 0.12	3.04 ± 0.08	2.93 ± 0.31	3.09 ± 0.26	n.s.
		3/5	3.14 ± 0.22 ab	2.18 ± 0.68 b	3.52 ± 1.06 a	3.14 ± 0.95 ab	2.77 ± 0.42 ab	2.84 ± 0.47 ab	***
	2021	9/4	3.73 ± 0.74	2.91 ± 1.22	2.82 ± 0.49	3.35 ± 0.29	2.76 ± 0.25	2.97 ± 0.60	n.s.
		18/4	3.74 ± 1.13 abc	2.78 ± 0.45 bc	2.75 ± 0.53 c	3.96 ± 0.73 a	2.78 ± 0.26 bc	3.88 ± 1.03 ab	***
		27/4	2.46 ± 0.14 ab	2.47 ± 0.05 ab	2.49 ± 0.11 ab	2.51 ± 0.10 ab	2.42 ± 0.08 b	2.57 ± 0.11 a	**
Chl_(a+b)_/Car	2019	14/4	3.84 ± 0.35 c	4.21 ± 0.22 bc	4.21 ± 0.24 bc	4.66 ± 0.17 a	4.11 ± 0.41 c	4.55 ± 0.31 ab	***
		22/4	4.41 ± 0.59 ab	4.57 ± 0.65 ab	4.21 ± 0.23 b	4.13 ± 0.43 b	4.75 ± 0.27 a	4.57 ± 0.32 ab	***
		3/5	4.27 ± 0.28 b	4.75 ± 0.84 ab	4.13 ± 0.29 b	5.26 ± 0.79 a	4.09 ± 0.41 b	4.80 ± 0.32 ab	**
	2021	9/4	4.01 ± 0.29 c	5.08 ± 0.96 ab	4.51 ± 0.79 abc	4.26 ± 0.42 bc	4.63 ± 0.60 abc	5.39 ± 0.74 a	**
		18/4	4.34 ± 0.92	5.35 ± 0.91	4.47 ± 0.56	4.45 ± 0.85	4.98 ± 0.71	5.46 ± 0.62	n.s.
		27/4	5.41 ± 0.29 b	5.47 ± 0.31 b	5.62 ± 0.34 b	5.94 ± 0.51 ab	5.72 ± 0.39 b	6.31 ± 0.38 a	***

Data are presented as mean ± SD of eight replicates. Different lowercase letters represent significant differences between treatments. The absence of letters indicates no significant differences between treatments (n.s., not significant). Asterisks represent significant differences (***p ≤ 0.001; **p ≤ 0.01).

**Table 6:** Total chlorophyll [Chl_(a+b_)], carotenoids (Car), and related ratios [Chl_a_/Chl_b_, Chl_(a+b)/_Car] of cv. *Lapins* leaves in 2019 and 2021.

cv. *Lapins*		Date	C	EM 0.15%	EM 0.30%	GB 0.25%	GB 0.40%	Mix	p
Chl_(a+b)_ (µg g^−1^ DW)	2019	14/4	3.13 ± 1.54 b	3.25 ± 0.23 b	3.58 ± 1.02 b	5.19 ± 0.91 a	3.78 ± 0.79 ab	4.58 ± 1.07 ab	***
		22/4	3.33 ± 0.23 c	3.80 ± 1.11 bc	3.35 ± 0.38 c	5.41 ± 0.60 a	3.92 ± 0.69 bc	4.69 ± 0.94 ab	***
		3/5	3.42 ± 0.23 c	3.66 ± 0.79 bc	3.45 ± 0.34 c	5.64 ± 0.50 a	4.32 ± 0.36 b	5.33 ± 0.48 a	***
	2021	9/4	2.15 ± 0.53 c	2.24 ± 0.40 c	2.47 ± 0.35 bc	3.00 ± 0.47 ab	3.02 ± 0.34 ab	3.70 ± 0.66 a	***
		18/4	2.32 ± 0.49 c	2.47 ± 0.28 c	2.64 ± 0.44 bc	3.02 ± 0.41 b	3.13 ± 0.30 b	3.94 ± 0.23 a	***
		27/4	2.89 ± 0.41 b	3.43 ± 0.74 ab	3.41 ± 0.69 ab	4.14 ± 0.60 a	3.20 ± 0.48 b	3.31 ± 0.63 ab	**
Car (µg g^−1^ DW)	2019	14/4	0.82 ± 0.41	0.77 ± 0.08	0.84 ± 0.19	1.11 ± 0.17	0.93 ± 0.24	1.00 ± 0.18	n.s.
		22/4	0.76 ± 0.10 c	0.85 ± 0.27 bc	0.79 ± 0.07 bc	1.14 ± 0.12 a	0.96 ± 0.21 abc	1.02 ± 0.14 ab	***
		3/5	0.79 ± 0.21 b	0.80 ± 0.06 b	0.84 ± 0.05 b	1.09 ± 0.15 a	1.06 ± 0.11 a	1.11 ± 0.08 a	***
	2021	9/4	0.45 ± 0.10 c	0.70 ± 0.17 a	0.56 ± 0.10 abc	0.70 ± 0.06 a	0.66 ± 0.08 ab	0.53 ± 0.10 bc	***
		18/4	0.47 ± 0.09 c	0.73 ± 0.05 a	0.60 ± 0.05 abc	0.69 ± 0.10 ab	0.63 ± 0.07 ab	0.58 ± 0.15 bc	***
		27/4	0.49 ± 0.05 b	0.62 ± 0.11 ab	0.61 ± 0.11 ab	0.66 ± 0.11 a	0.56 ± 0.07 ab	0.61 ± 0.13 ab	**
Chl_a_/Chl_b_	2019	14/4	3.03 ± 0.11	3.03 ± 0.20	3.07 ± 0.13	3.09 ± 0.16	3.01 ± 0.21	3.09 ± 0.26	n.s.
		22/4	3.04 ± 0.51	2.77 ± 0.91	3.08 ± 0.12	3.04 ± 0.08	2.93 ± 0.31	3.09 ± 0.26	n.s.
		3/5	3.14 ± 0.22 ab	2.18 ± 0.68 b	3.52 ± 1.06 a	3.14 ± 0.95 ab	2.77 ± 0.42 ab	2.84 ± 0.47 ab	***
	2021	9/4	3.73 ± 0.74	2.91 ± 1.22	2.82 ± 0.49	3.35 ± 0.29	2.76 ± 0.25	2.97 ± 0.60	n.s.
		18/4	3.74 ± 1.13 abc	2.78 ± 0.45 bc	2.75 ± 0.53 c	3.96 ± 0.73 a	2.78 ± 0.26 bc	3.88 ± 1.03 ab	***
		27/4	2.46 ± 0.14 ab	2.47 ± 0.05 ab	2.49 ± 0.11 ab	2.51 ± 0.10 ab	2.42 ± 0.08 b	2.57 ± 0.11 a	**
Chl_(a+b)_/Car	2019	14/4	3.84 ± 0.35 c	4.21 ± 0.22 bc	4.21 ± 0.24 bc	4.66 ± 0.17 a	4.11 ± 0.41 c	4.55 ± 0.31 ab	***
		22/4	4.41 ± 0.59 ab	4.57 ± 0.65 ab	4.21 ± 0.23 b	4.13 ± 0.43 b	4.75 ± 0.27 a	4.57 ± 0.32 ab	***
		3/5	4.27 ± 0.28 b	4.75 ± 0.84 ab	4.13 ± 0.29 b	5.26 ± 0.79 a	4.09 ± 0.41 b	4.80 ± 0.32 ab	**
	2021	9/4	4.01 ± 0.29 c	5.08 ± 0.96 ab	4.51 ± 0.79 abc	4.26 ± 0.42 bc	4.63 ± 0.60 abc	5.39 ± 0.74 a	**
		18/4	4.34 ± 0.92	5.35 ± 0.91	4.47 ± 0.56	4.45 ± 0.85	4.98 ± 0.71	5.46 ± 0.62	n.s.
		27/4	5.41 ± 0.29 b	5.47 ± 0.31 b	5.62 ± 0.34 b	5.94 ± 0.51 ab	5.72 ± 0.39 b	6.31 ± 0.38 a	***

Data are presented as mean ± SD of eight replicates. Different lowercase letters represent significant differences between treatments. The absence of letters indicates no significant differences between treatments (n.s., not significant). Asterisks represent significant differences (***p ≤ 0.001; **p ≤ 0.01).

In almost all situations, C leaves from cv. *Early Bigi* (**Table 5**) exhibited lower values of total chlorophyll and carotenoids, with an inverse behavior recorded mainly for GB 0.25% and Mix treatments. The Chl_a_/Chl_b_ and Chl(_a+b_)/Car ratios reflect the variations in the individual components. For cv. *Lapins* (**Table 6**), this pattern is also evident, even though other treatments present statistically similar values to those of the C treatment.

This increase in photosynthetic pigments after the application of biostimulants has been previously documented in sweet cherry (Correia et al., 2020b; Serapicos et al., 2022) and in other plants (Yakhin et al., 2017; Afonso et al., 2022). This increase may be attributed to biostimulants reducing the amount of pigment loss due to peroxidation and pigment decomposition by oxygen radicals or by boosting pigment biosynthesis (Navari-Izzo et al., 1990; Hojjati et al., 2023).

Other possible explanations for the observed effects include improved water and ion use efficiency, enhanced stomatal conductance, increased photosynthetic capacity, and the presence of bioactive compounds (Ali et al., 2020; Bahmani Jafarlou et al., 2022; Jafarlou et al., 2022). These factors contribute to inducing sink capability by facilitating the supply and translocation of photoassimilates from leaves to other parts of the plant (Arif et al., 2023).

The Chl_a_/Chl_b_ ratio has been reported to vary from 2 to 4, depending on the plant (Filimon et al., 2016), and serves as an indicator of functional pigment equipment and photosynthetic apparatus light adaptation (Lichtenthaler and Buschmann, 2005). For both studied cultivars, all recorded values were above 2, and higher Chl_a_/Chl_b_ values are often related to a greater capacity to absorb light and consequently higher photosynthetic rates. However, this ratio can be affected by the growth habit of trees, where low light microenvironment can point to an increase in light-harvesting complexes of photosystem II and consequently a decrease of Chl_a_/Chl_b_ (Demmig-Adams, 1998).

The chlorophyll - to-carotenoid ratio (Chl_total_/Car_total_) was also influenced by the studied biostimulants, with a more pronounced effect observed for cv. *Early Bigi* (**Table 5**). Carotenoids not only are considered accessory pigments but also play an essential role in photoprotection by accepting energy from chlorophyll and dissipating it as heat, thus preventing chloroplast and tissue damage (Hashimoto et al., 2016). Their increase in the use of biostimulants is well-documented (Mones Sardrodi et al., 2022), including in sweet cherries, as recently demonstrated by Correia et al. (2020b). The lower values of Chl_total_/Car_total_ might indicate that trees were under higher stress, with samples showing higher values probably more able to be protected by carotenoids against photooxidation (Barthod et al., 2007; Loggini et al., 1999).

#### 3.3.2 Total soluble sugars and starch

**Figure 4:**
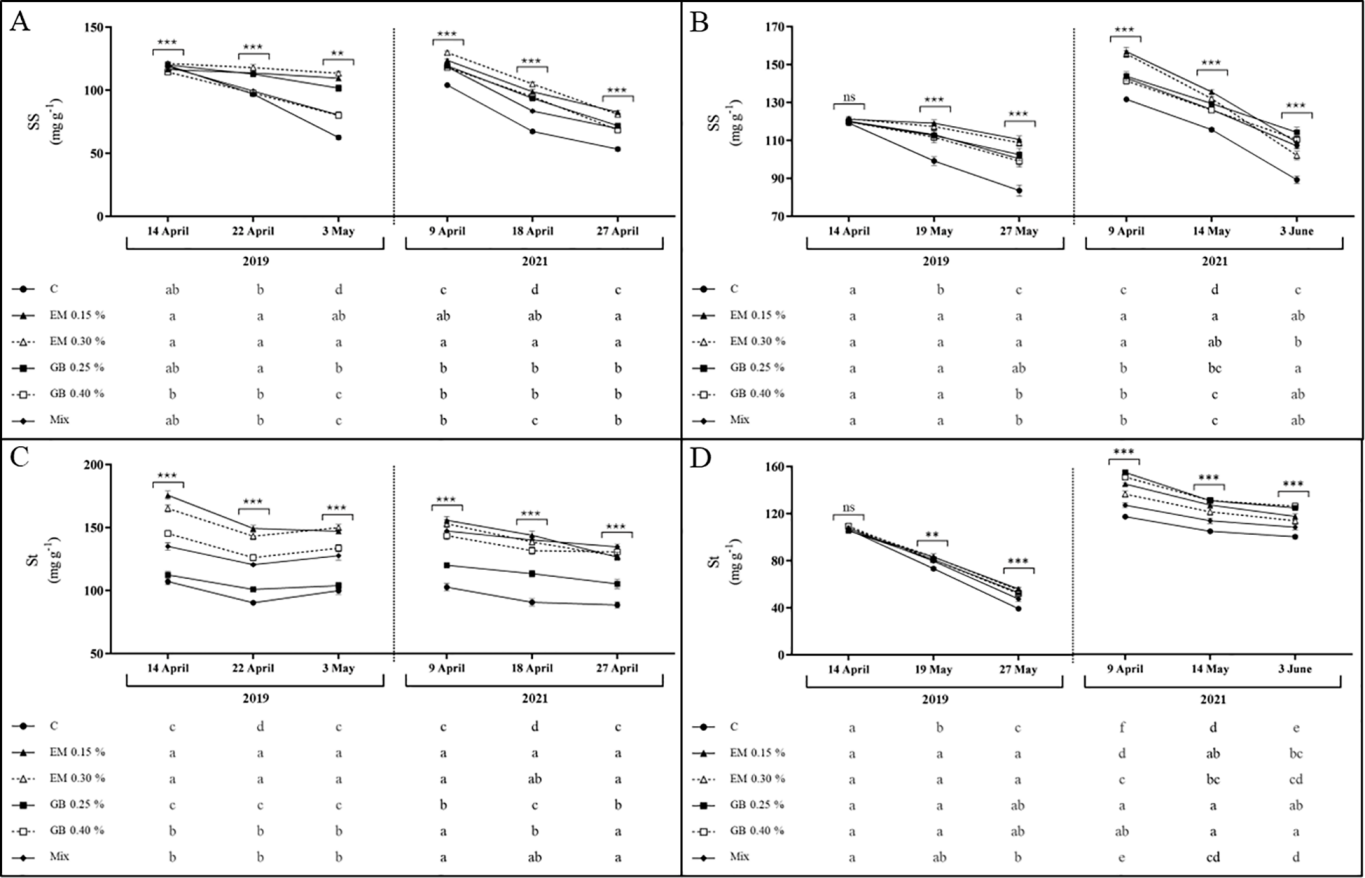
Soluble sugars (SS, mg g^−1^ DW) and starch contents (St, mg g^−1^ DW) of cvs. *Early Bigi* **(A, C)** and *Lapins* **(B, D)** leaf cherry after spray treatment application in 2019 and 2021. Data are mean ± SD of eight replicates. Different lowercase letters represent significant differences. The absence of letters indicates no significant differences between treatments (n.s., not significant). Asterisks represent significant differences (***p ≤ 0.001; **p ≤ 0.01).

Soluble sugars and starch content present, for both cultivars and across both years, a very similar trend, with a decrease in their content as fruit development progressed (**Figure 4**). Moreover, their content was clearly influenced by the application of biostimulants, year, phenological stage, and the interactions between all the factors (apart from the interaction of treatment, year, and phenological stage in cv. *Early Bigi* and interaction of treatment and phenological stage of cv. *Lapins*, regarding starch content) (**Supplementary Table S1**). For soluble sugars (SS) (**Figure 4**), lower contents were observed in the control (C) treatment, a trend also recorded for starch content. These metabolites are generally associated with responses to different types of stress (Thalmann and Santelia, 2017), with soluble sugars being osmoprotectants (Chaves et al., 2002), while starch is mostly a reservoir for future use, depending on the source–sink dynamics (Brito et al., 2018). Overall, higher values were recorded for EM-treated leaves. These higher values might be linked to higher photosynthetic activity in leaves treated with biostimulants, leading to increased production of sugars and starch (Correia et al., 2020b; Serapicos et al., 2022). Additionally, biostimulant application can upregulate gene expression associated with carbohydrate metabolism (Contartese et al., 2016). Nonetheless, carbohydrate dynamics is very complex, influenced not only by plant growth conditions but also by genotype and even the specific types of sugars involved. Some sugars, such as sucrose and glucose, function as osmolytes or are involved in cellular respiration, while others (fructose) are crucial for secondary metabolite synthesis (Rosa et al., 2009). The key differences observed between C and treated samples are a lower amount of reduction (starch in 2019, in cv. *Lapins*, or sugars, in 2019, for both cultivars) or an increased initial content. However, the overall decreases in sugars and starch content in leaves during fruit growth have been previously recorded (Quentin et al., 2013), influenced by the presence of fruit, which indicates that sweet cherry fruits are strong sinks (Falchi et al., 2020).

#### 3.3.3 Total soluble protein

**Figure 5:**
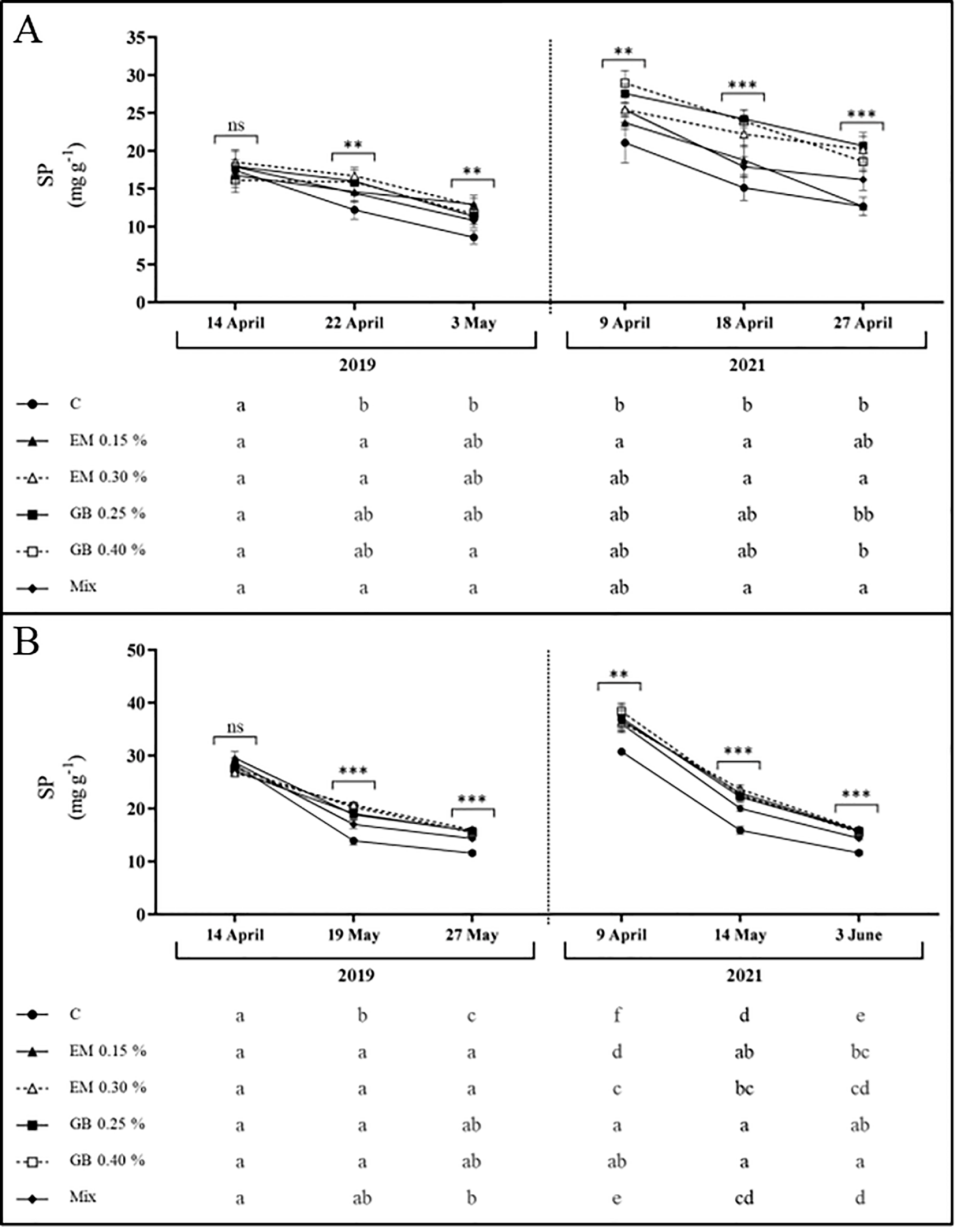
Total soluble protein content (SP, mg g^−1^ DW) of cvs. *Early Bigi* **(A)** and *Lapins* **(B)** leaf cherry after spray treatment application in 2019 and 2021. Data presented are mean ± SD of eight replicates. Different lowercase letters represent significant differences. The absence of letters indicates no significant differences between treatments (n.s., not significant). Asterisks represent significant differences (***p ≤ 0.001; **p ≤ 0.01).

Soluble protein content (SP) follows a similar pattern to that of carbohydrates, with lower values in C treatment across both years and cultivars (**Figure 5**). In leaves of both cultivars, the concentration of total soluble protein was affected by treatment (*p* ≤ 0.001), year (*p* ≤ 0.001), and phenological stage (*p* ≤ 0.001). Furthermore, the interaction between treatment and year (*p* ≤ 0.001) for cv. *Early Bigi* and the interaction of treatment and phenological stage (*p* ≤ 0.001) and the interaction year and phenological stage (*p* ≤ 0.001) for cv. *Lapins* also influenced the total soluble protein content (**Supplementary Table S1**).

The accumulation of proteins after the foliar application of biostimulants has been recorded elsewhere (Yakhin et al., 2017; Afonso et al., 2022). This increase may be associated with the rich bioactive endogenous profile of seaweed, which includes hormones, minerals, and vitamins that promote enzymatic actions responsible for protein synthesis (Aslam et al., 2016), and is linked to the increased expression of genes (Goñi et al., 2018; Baltazar et al., 2021) or the N input present in the biostimulants (Afonso et al., 2022). Additionally, the amino acid glycine, a reduced form of nitrogen, can be directly assimilated by leaves, thereby accelerating protein biosynthesis (Zargar Shooshtari et al., 2020). On the other hand, the reduction of protein content with fruit development may possibly be related to the onset of mobilization of foliar N towards other plant parts (Thimann, 1980).

#### 3.3.4 Total phenolics

**Figure 6:**
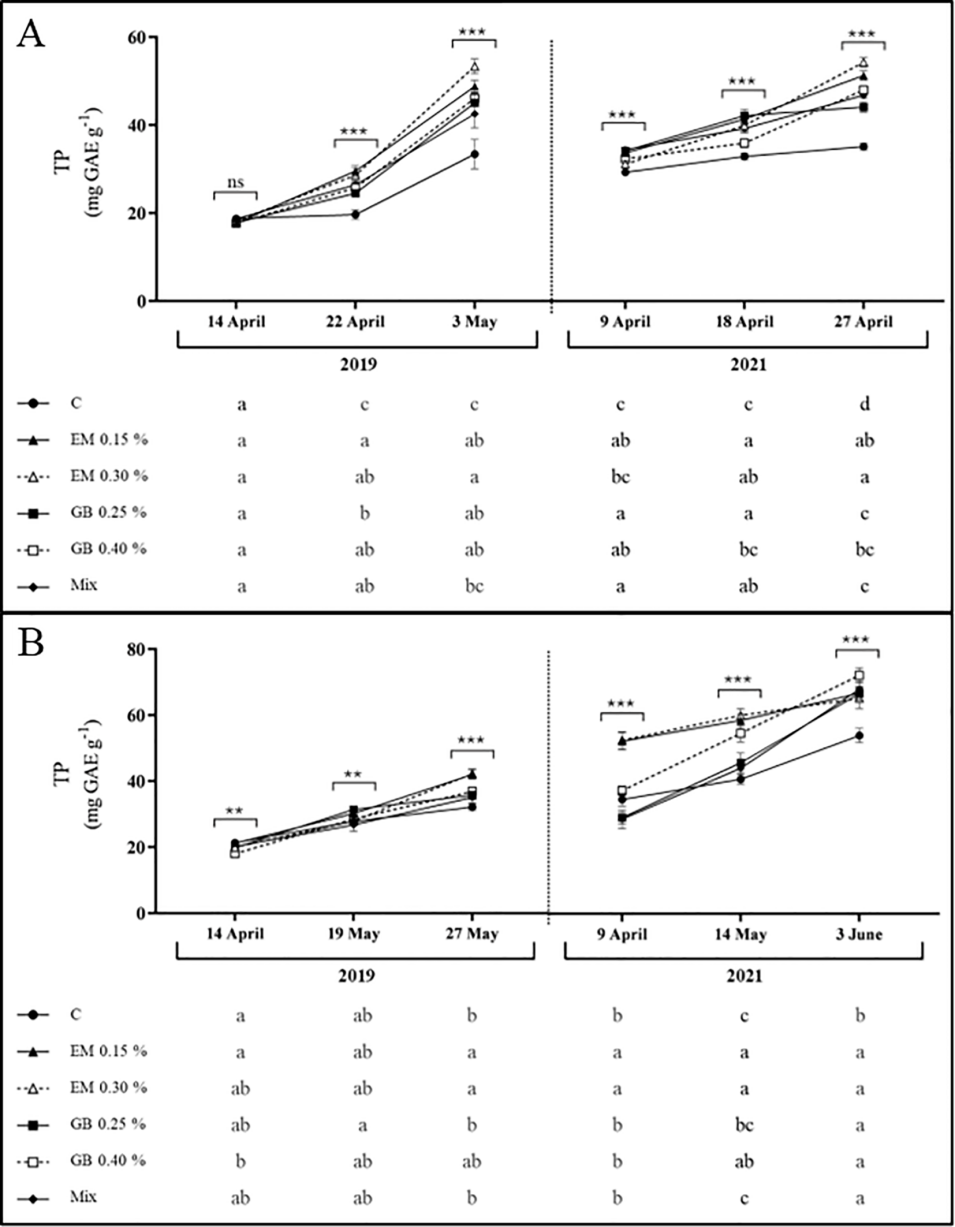
Total phenolics content (TP, mg GAE g^−1^ DW) of cvs. *Early Bigi* **(A)** and *Lapins* **(B)** leaf cherry after spray treatment application in 2019 and 2021. Data presented are mean ± SD of eight replicates. Different lowercase letters represent significant differences. The absence of letters indicates no significant differences between treatments (n.s., not significant). Asterisks represent significant differences (***p ≤ 0.001; **p ≤ 0.01).

Data for total phenolic content show an increase in all situations as fruit development progresses (**Figure 6**). This pattern is commonly observed in plants (Colaric et al., 2006), including sweet cherries (Serapicos et al., 2022), and the average value is within the range found in sweet cherry leaves (Gonçalves et al., 2008; Dziadek et al., 2019; Nunes et al., 2021). In both cultivars, the effect of biostimulants was evident (*p* ≤ 0.001), as well as the effect of the remaining factors. However, only observed in cv. *Early Bigi*, the interactions between treatment and year (*p* > 0.05) and between treatment, year, and phenological stage (*p* > 0.05) did not significantly affect the total phenolic content (**Supplementary Table S1**). Overall, the greatest results were recorded with algae-based biostimulants, regarding cv. *Early Bigi*, although differences can be observed with all treatments. For cv. *Lapins*, algae-based biostimulants also led to increased phenolic content, but with reduced variations when compared to other treatments. The increased content of phenolic compounds in spray samples might be linked to the ability of biostimulants to enhance the activity of key enzymes, like phenylalanine ammonia lyase and tyrosine aminotransferase, that are involved in phenolics biosynthesis (Kulkarni et al., 2019; Afonso et al., 2022). On the other hand, it may be attributed to the improved activity of endogenous antioxidant enzymes, hence protecting existing phenolics form oxidation (Pylak et al., 2019; Afonso et al., 2022).

### 3.4 Cell membrane damage

Biotic or abiotic stresses can cause the accumulation of reactive oxygen species, causing damage to cell membranes through lipid peroxidation, thereby changing their permeability (Sachdev et al., 2021). These changes in permeability can be monitored by measuring the EL, an indicator of cell membrane integrity (Bajji et al., 2001), or by detecting the presence of TBARS.

**Figure 7:**
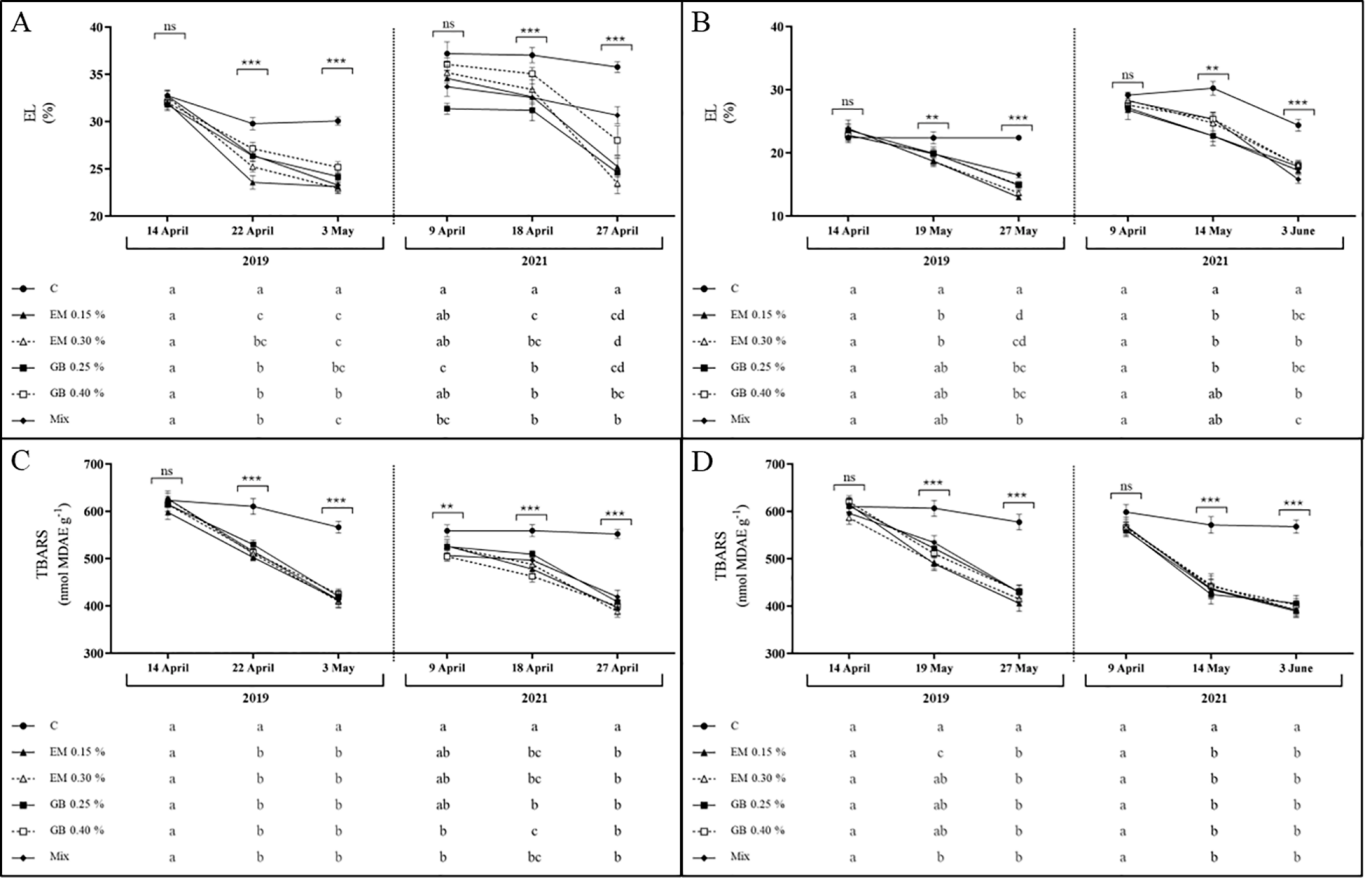
Electrolyte leakage (EL, %) and thiobarbituric acid reactive substances concentration (TBARS, nmol MDAE g^−1^ DW) of cvs. *Early Bigi* **(A, C)** and *Lapins* **(B, D)** leaf cherry after spray treatment application in 2019 and 2021. Data are mean ± SD of eight replicates. Different lowercase letters represent significant differences. The absence of letters indicates no significant differences between treatments (n.s., not significant). Asterisks represent significant differences (***p ≤ 0.001; **p ≤ 0.01; *p ≤ 0.05).

In the current study, both indexes of membrane damage exhibit a very similar tendency (**Figure 7**). In leaves of both cultivars, these indexes were affected by year (*p* ≤ 0.001), phenological stage (*p* ≤ 0.001), treatments (*p* ≤ 0.001), and the interaction of treatment and phenological stage (*p* ≤ 0.001). The EL was also influenced by the interaction between year and treatment (*p* ≤ 0.001) and the interaction between treatment, year, and phenological stage (*p* ≤ 0.01) only for cv. *Early Bigi*. The interaction between year and phenological stage affected the EL (*p* ≤ 0.001 for both cultivars) and TBARS (*p* ≤ 0.001 and *p* ≤ 0.01, for cv. *Early Bigi* and *Lapins*, respectively) (**Supplementary Table S1**).

Results for cv. *Early Bigi* (**Figure 7**) in both years show a similar trend, with an overall decrease in EL with the advance of fruit development, observed in both 2019 and 2021. The application of biostimulants appears to affect this specific parameter, as significantly higher values were recorded for C leaves in all sampling dates of both years (the only exception on the first sampling of 2019). This same pattern was recorded in leaves of cv. *Lapins*, with a decrease of EL with fruit development, and higher values were recorded for C treatment (with exceptions noted in the first sampling of both years).

The lower EL observed with the application of biostimulants might point out a positive effect with the use of these compounds in preserving cell membranes during dehydration (Savé et al., 1999), even in the absence of severe stress, as in the current work.

The evaluation of TBARS allows an overview regarding peroxidation of membrane lipids mediated by reactive oxygen species, which can lead to cellular damage (Sofo et al., 2004; Beis and Patakas, 2012), and typically, higher TBARS values indicate a higher exposure to stress. The results of the present work underscore the significant effect of the use of biostimulants in this specific parameter (**Figure 7**). The overall trend of TBARS is a decrease of their content with fruit development, in all situations. However, the use of biostimulants presented two different effects: firstly, in all samples, the decrease in TBARS content was significantly higher than that recorded in the C samples; in addition, in the 2021 dataset, the initial TBARS content was already considerably lower in leaves from sprayed trees. This pronounced effect of biostimulants on TBARS data has previously been recorded in sweet cherry leaves (Correia et al., 2020b).

### 3.5 Conclusion

This study evaluated the impact of biostimulants on leaf water status, photosynthetic pigments, soluble sugars, starch, soluble proteins, and leaf gas exchange parameters in two cultivars of sweet cherries.

Results revealed significant differences between cultivars and years, with notable effects observed in 2021, a hotter and dryer year compared to 2019. Despite variations, the application of biostimulants generally led to an improved leaf water status, enhanced photosynthetic pigment content, and increased photosynthetic activity (Jolayemi et al., 2023). Furthermore, the application of biostimulants also contributed to the preservation of cell membrane integrity.

These improvements were most evident after the application of both concentrations of the seaweed-based biostimulant *E. maxima* and treatment GB 0.40%, which positively influenced the performance of cherry trees.

These findings indicate the potential of biostimulants to mitigate the impact of environmental stressors and enhance physiological processes in sweet cherry cultivation, contributing to improved crop performance. Further research is warranted to elucidate the underlying mechanisms and optimize biostimulant applications for sustainable cherry production.

## Data Availability

The raw data supporting the conclusions of this article will be made available by the authors, without undue reservation.
