# Supplementary material for: GLYCINE betaine and seaweed-based biostimulants improved leaf water status and enhanced photosynthetic activity in sweet cherry trees
Source: Front Plant Sci. 2024 Dec 20;15:1467376. doi: 10.3389/fpls.2024.1467376 (PMC11695132; doi:10.3389/fpls.2024.1467376)
Supplement: Supplementary file 1 [file Table1.docx]

**Table S1**- Statistical analysis of Treatment (T), Year (Y), and Phenological Stage (PS) effects and interactions on studied leaf parameters.

| Leaf parameters | Cultivar | *P* (T) | *P* (Y) | *P* (PS) | *P* (T*Y) | *P* (T*PS) | *P* (Y*PS) | *P* (T*Y*PS) |
| --- | --- | --- | --- | --- | --- | --- | --- | --- |
| RWC | *Early Bigi* | ≤ 0.001 | > 0.05 | ≤ 0.001 | ≤ 0.001 | ≤ 0.01 | ≤ 0.001 | > 0.05 |
|  | *Lapins* | ≤ 0.001 | ≤ 0.01 | ≤ 0.001 | ≤ 0.001 | ≤ 0.001 | ≤ 0.001 | ≤ 0.05 |
| LMA | *Early Bigi* | ≤ 0.001 | ≤ 0.001 | ≤ 0.001 | ≤ 0.001 | ≤ 0.001 | ≤ 0.01 | > 0.05 |
|  | *Lapins* | ≤ 0.001 | ≤ 0.001 | ≤ 0.001 | > 0.05 | ≤ 0.01 | ≤ 0.001 | > 0.05 |
| Chl_(a+b)_ | *Early Bigi* | ≤ 0.001 | ≤ 0.001 | ≤ 0.001 | ≤ 0.001 | > 0.05 | > 0.05 | ≤ 0.01 |
|  | *Lapins* | ≤ 0.001 | ≤ 0.001 | ≤ 0.001 | ≤ 0.01 | ≤ 0.001 | ≤ 0.01 | ≤ 0.001 |
| Car | *Early Bigi* | ≤ 0.001 | ≤ 0.001 | > 0.05 | ≤ 0.001 | > 0.05 | > 0.05 | > 0.05 |
|  | *Lapins* | ≤ 0.001 | > 0.05 | ≤ 0.01 | > 0.05 | ≤ 0.001 | > 0.05 | > 0.05 |
| Chl_a_/Chl_b_ | *Early Bigi* | ≤ 0.001 | > 0.05 | ≤ 0.001 | ≤ 0.01 | ≤ 0.01 | ≤ 0.001 | ≤ 0.01 |
|  | *Lapins* | ≤ 0.001 | ≤ 0.001 | > 0.05 | > 0.05 | ≤ 0.01 | ≤ 0.01 | > 0.05 |
| Chl_(a+b)_/Car | *Early Bigi* | ≤ 0.001 | ≤ 0.001 | ≤ 0.001 | ≤ 0.001 | > 0.05 | ≤ 0.001 | > 0.05 |
|  | *Lapins* | ≤ 0.01 | ≤ 0.01 | ≤ 0.001 | > 0.05 | > 0.05 | > 0.05 | > 0.05 |
| SS | *Early Bigi* | ≤ 0.001 | ≤ 0.001 | ≤ 0.001 | ≤ 0.001 | ≤ 0.001 | ≤ 0.001 | ≤ 0.001 |
|  | *Lapins* | ≤ 0.001 | ≤ 0.001 | ≤ 0.001 | ≤ 0.01 | ≤ 0.001 | ≤ 0.001 | ≤ 0.001 |
| St | *Early Bigi* | ≤ 0.001 | ≤ 0.01 | ≤ 0.001 | ≤ 0.001 | ≤ 0.001 | ≤ 0.001 | > 0.05 |
|  | *Lapins* | ≤ 0.001 | ≤ 0.001 | ≤ 0.001 | ≤ 0.001 | > 0.05 | ≤ 0.001 | ≤ 0.001 |
| SP | *Early Bigi* | ≤ 0.001 | ≤ 0.001 | ≤ 0.001 | ≤ 0.001 | > 0.05 | > 0.05 | > 0.05 |
|  | *Lapins* | ≤ 0.001 | ≤ 0.001 | ≤ 0.001 | > 0.05 | ≤ 0.05 | ≤ 0.001 | > 0.05 |
| TP | *Early Bigi* | ≤ 0.001 | ≤ 0.001 | ≤ 0.001 | > 0.05 | ≤ 0.001 | ≤ 0.05 | > 0.05 |
|  | *Lapins* | ≤ 0.001 | ≤ 0.001 | ≤ 0.001 | ≤ 0.001 | ≤ 0.01 | ≤ 0.001 | ≤ 0.001 |
| EL | *Early Bigi* | ≤ 0.001 | ≤ 0.001 | ≤ 0.001 | ≤ 0.001 | ≤ 0.001 | ≤ 0.001 | ≤ 0.01 |
|  | *Lapins* | ≤ 0.001 | ≤ 0.001 | ≤ 0.001 | > 0.05 | ≤ 0.001 | ≤ 0.001 | > 0.05 |
| TBARS | *Early Bigi* | ≤ 0.001 | ≤ 0.001 | ≤ 0.001 | > 0.05 | ≤ 0.001 | ≤ 0.001 | > 0.05 |
|  | *Lapins* | ≤ 0.001 | ≤ 0.001 | ≤ 0.001 | > 0.05 | ≤ 0.001 | ≤ 0.01 | > 0.05 |
